# Male infertility is not liked with HSF1, HSF2 and UBE2I gene polymorphisms among Indian subjects

**DOI:** 10.6026/97320630017715

**Published:** 2021-08-31

**Authors:** Pravin Kumar Gangwar, Satya Narayan Sankhwar, Shriya Pant, Bhupendra Pal Singh, Abbas Ali Mahdi, Rajender Singh

**Affiliations:** 1 Department of Urology, King George's Medical University, Lucknow, Uttar Pradesh, India; 2 Department of Biochemistry, King George's Medical University, Lucknow, Uttar Pradesh, India; 3 Division of Endocrinology, Central Drug Research Institute, Lucknow, Uttar Pradesh, India

**Keywords:** Heat shock factor genes, Ubiquitin conjugating enzyme E2I, Single nucleotide polymorphism, Male infertility

## Abstract

We analysed the polymorphisms at rs78202224 (C/A) for HSF1 gene, rs139496713 (C/T) and rs45504694 (C/A) for HSF2 gene and rs116868327 (G/A) for UBE2I gene in 547 infertile cases (non-obstructive azoospermia = 464, asthenozoospermia = 83) and 419 proven fertile controls of similar age group and ethnicity. SNP genotyping was done using AgenaMassARRY platform (Agena Bioscience, CA). Common, heterozygous, rare genotypes and allelic frequencies were analysed using dominant, recessive and co-dominant models. Data shows no significant association between HSF1, HSF2 polymorphisms and male infertility. However, under dominant (GG vs GA+AA) and co-dominanat (GG vs GA) model, polymorphism at the rs116868327 (G/A) locus in UBE2I gene was found to be linked with asthenozoospermia in males with a significant odd-ratio of 6.91 (confidence interval at 95% was 1.52-31.46; p=0.017). Moreover, frequency of rare allele was higher (2.4%) compared to controls (0.4%). Thus, this data showed a significant risk of developing asthenozoospermic condition in males (Odds ratio= 6.75; Confidence interval at 95%= 1.50-30.49; P= 0.018]. Hence, more number of genotyping studies along with the functional assay in multiple cohorts is needed to validate potential variants associated with male infertility.

## Background:

Heat shock factor 1 (HSF1) is required for the response against cellular stress and known as the primary transcription factor, while HSF2 is also involved in the regulation of heat shock protein expression to support cells against environmental stresses as well as in cellular processes and spermatogenesis [1]. Active form of HSF1 is responsible for the disturbances in spermatogenesis may be due to the mutations/ polymorphisms in this gene. It affects the spermatogenesis and causes the death of spermatocytes at pachytene stage where HSF1 remain present in an active state which could be a sign of accretion of defected proteins and induced cell death [2,3]. Whereas, HSF1 null mouse, having a complete loss of its activity displayed normal fertility. It also plays an important task by protecting immature germ cells along with spermatogonia against testis hyperthermia [4]. Taken together, HSF1 plays two opposite roles in spermatogenesis and can be involved in quality control of male germ cells [2-4]. Interestingly, loss of HSF2 activity resulted in a phenotype with low testicular volume and sperm count with increased apoptosis. Further, complete loss of both HSF1and HSF2 gene function promotes severe defects in spermatogenesis causing sterility [5]. These data indicate the importance of the transcriptional activity of both HSF1 and HSF2 for normal spermatogenesis. UBE2I (Ubiquitin Conjugating Enzyme E2I) gene encodes a SUMO-conjugating enzyme UBC9 in humans, mainly expressed in heart, pancreas, kidney, liver, lung, skeletal muscle, placenta, brain and in testis as well. It plays an important role in sumoylation and ubiquitination processes. Recently, sumoylation has emerged as a crucial regulator of proteins with significant roles in spermatogenesis [6]. Rogers et al. (2004) initially identified SUMO proteins in XY body of pachytene spermatocytes in rat and suggested the crucial function of sumoylation in spermatogenesis [7]. Several experimental studies suggest the significance of SUMO modifications in meiosis and spermatid elongation [6,8]. Above data indicates the role of different SUMO isoforms in protein modifications during germ cell development. On the other hand excessive sumoylation has been observed as a potential marker of defective sperms in human [6]. Therefore, we may suggest an important role of UBE2I gene in spermatogenesis via SUMO conjugation pathway. These genes were selected based on their known expression and function in spermatogenesis/ spermiogenesis. However, data on genetic variations in HSF1, HSF2 and UBE2I genes for infertility in males is scanty. A single study on HSF2 gene in this context is known [9]. Therefore, it is of interest to document data on the link between male infertility and HSF1, HSF2 and UBE2I gene polymorphisms.

## Materials & Methods:

### Study Population

The study was done on the patients of Non-obstructive azoospermia (NOA) and Asthenozoospermia excluding obstructive azoospermia. The study was approved from the Institutional Ethics Committee, K.G.M.U, Lucknow, U.P, India (Letter no.1163/R-Cell/12, Dated-14/05/2012). All the participants were enrolled from the Department of Urology, King George’s Medical University (K.G.M.U), Lucknow, U.P, India, after obtaining their informed written consent. All the study participants belonged to the Indo-European ethnicity. All the subjects underwent detailed medical and physical examinations before sample collection. The case group consists of infertile men aged 21 to 40 years, having infertility more than one year with a normal fertile female partner to avoid any possibility of involvement of female factors. Further, the subjects with endocrine abnormalities, acquired and congenital structural defects of the urogenital system (cystic fibrosis, Young’s syndrome, etc.), karyotype abnormalities showing chromosomal defects and patients with any history of surgery of genital tract obstruction/dysfunction (varicocele, obstructive azoospermia) were excluded. The infertile individuals with excessive alcoholism, smoking, drug abuse (ecstasy, marijuana and recreational substances) and having any history of radiotherapy and chemotherapy were also excluded. The patient group, after following the above inclusion and exclusion criteria, comprised of 547 infertile individuals. Semen sample of all the patients was collected by masturbation after an abstinence period of 3-5 days. Semen parameters were analysed following the criteria of the World Health Organization (WHO), (2010) [10]. The patients were distributed into azoospermia (absence of mature sperm in semen, n = 464) and asthenozoospermia (progressive sperm motility < 32% and sperm count ≥ 15 million/ml, n = 83). In control group, 419 proven fertile men were enrolled, belonged to the same age-group (21-40 years) and ethnicity as that of the cases, who had fathered a child during the last three years without having history of any sexual abnormality. All the control samples were collected from the individuals visiting the urology OPD for problems other than infertility. We also collected 5 ml blood sample from all the study participants for DNA isolation and further genotyping experiments.

### SNP Genotyping:

SNP genotyping was performed using AgenaMassARRY platform (Agena Bioscience, CA). The MassARRY system is non-fluorescent detection uses mass spectrometry to accurately measure polymerase chain reaction (PCR)-derived amplicons. It has high capability of multiplexing up to around 40plex from a single well. In brief, genotyping perform in two steps of PCR reactions; In the first step, a locus-specific PCR was run to amplify the DNA stretch containing the polymorphic site. In the second step, a single base extension was performed using mass modified dideoxy nucleotide terminators of oligonucleotide primer, which annealed immediately upstream of the polymorphic site of interest. Further, the distinct mass of the extended primer was identified as the SNP allele using MALDI-TOF mass spectrometry.

### Statistical Analysis:

Statistical analysis was performed using the bio statistical tools online (http/www.vassarstats.net) and using different models such as genotypic, allelic, dominant, recessive and co-dominant models. Comparison of genotypes was performed by Fisher exact probability test. Statistical significance at p-value < 0.05 was considered as a significant difference.

## Results:

A total of 4 SNPs, a missense variant; rs78202224 (C/A) for HSF1 gene, a 3' UTR variant; rs139496713 (C/T) and a 5' UTR variant; rs45504694 (C/A) for HSF2 gene and one 3' UTR variant; rs116868327 (G/A) for UBE2I gene were selected based on their SIFT, PolyPhen and GMAF scores. We did large scale SNP genotyping in 966 samples. This cohort of samples included 419 fertile control samples and 547 infertile samples (NOA, n=464 and asthenozoospermia; n= 83). Average genotype calling in our study cohort was more than 95%, which depicted that genotyping was successful in 958 samples (545 cases and 413 controls) for rs78202224 (C/A) for HSF1 gene, 921 samples (527 cases and 394 controls) for rs139496713 (C/T), 943 samples (538 cases and 405 controls) for rs45504694 (C/A) for HSF2 gene and 946 samples (539 cases and 407 controls) for rs116868327 (G/A) for UBE2I gene. We found that all SNPs in our study cohort had minor allele frequency (MAF) more than or equal to 1%. MAF was ranging from 1% (rs116868327 (G/A) locus in UBE2I gene) to 18% (rs45504694 (C/A) locus in HSF2 gene). (Table 1 - see PDF)

The frequencies of genotypes CC, CA and AA for rs78202224 (C/A) for HSF1 gene in all infertile, azoospermic and asthenozoospermic cases were 71.4%, 26.6 % and 2.0%; 71.7%, 26.6% and 1.7% and 69.9%, 26.5% and 3.6 % and in controls it was 71.2%, 25.9% and 2.9%, respectively. (Table 2 - see PDF) Whereas, the genotype frequencies of CC, CT and TT for rs139496713 (C/T) for HSF2 gene in all infertile, azoospermic and asthenozoospermic individualss were found to be 96.8%, 3.2% and 0%; 96.2%, 3.8% and 0% and 100%, 0% and 0% while in controls it was 97.2%, 2.8% and 0%, respectively. (Table 3 - see PDF) In addition, frequencies of CC, CA and AA genotypes of rs45504694 (C/A) for HSF2 gene in all infertile, azoospermic and asthenospermic patients were observed as 62.8%, 37.2% and 0%; 62.3%, 37.7% and 0% and 65.9%, 34.1% and 0%, while in controls it was 64.2%, 35.6% and 0.2%, respectively. (Table 4 - see PDF) No significant association was observed between case and control group for variations at rs78202224 (C/A), rs139496713 (C/T) and rs45504694 (C/A) of HSF1 and HSF2 genes and susceptibility to infertility in males. Moreover, allelic frequencies of variations in HSF1 and HSF2 genes showed no significant differences between the two groups (p>0.05). On the other hand, genotypic distribution of GG, GA and AA genotypes for SNP (rs116868327, G/A) locus in UBE2I gene was 98.7%, 1.3% and 0% in all infertile patients; 99.3%, 0.7% and 0% in azoospermic group; 95.1%, 4.9% and 0% in asthenozoospermic group but in control group it was 99.3%, 0.7% and 0%, respectively. The genotypic analysis under dominant model (GG vs GA+AA) and co-dominanat/ additive model (GG vs GA) showed a significant association with asthenozoospermia [GG vs GA+AA: Odds ratio (95% Confidence interval) = 6.91 (1.52-31.46), P value = 0.017; GG vs GA: Odds ratio (95%Confidence interval) = 6.91 (1.52-31.46), P value = 0.017]. Moreover, rare allele frequency in asthenozoospermic group was higher than control group and it also showed a significant association with asthenozoospermia [Odds ratio (95%Confidence interval) = 6.75 (1.50-30.49), P value= 0.018]. (Table 5 - see PDF) 

## Discussion:

HSFs and UBE2I genes are linked in the gametogenesis in both genders [11,12]. Various experimental reports on mouse models have proven the important function of HSF1 and HSF2 genes in germ cell development in males while UBE2I gene in oocyte development in females [1-5,12]. To our surprise, we could find only two studies in humans that have looked into this aspect till now. First study by Mou et al. (2013) highlighted the association of genetic variants of HSF2 gene with idiopathic azoospermia (IA) in males while another study tried to explore the potential role of SNPs in HSF1 gene with human diseases [9,13]. Many genetic association studies have documented the potential impact of HSF1 and HSF2 on human health and diseases and tried to connect HSF1 gene variants and its altered levels, to schizophrenia, bipolar disorder, attention deficit hyperactivity disorder and breast cancer [14-17]. In the same way, low level of HSF2 mRNA was also observed in different types of malignancies in humans like invasive breast carcinoma, prostate carcinoma and various other carcinomas [16-18]. Moreover, UBE2I gene also plays an important role in progression of several cancers as lung, breast and bladder carcinoma [19,20].

It is of interest to explore the biological and clinical significance of genetic variants, this study evaluated the genetic polymorphisms of HSF1, HSF2 and UBE2I genes in human and tried to find out their involvement in the pathogenesis of male infertility. This study depicted the presence of rs78202224 variant (rare) genotype of HSF1 gene exclusively in 23 DNA samples out of 958 subjects. It suggest that the rare genotype frequency of this variant might be somewhat lower in our study population. Similarly, Bridges et al. (2015) found this variant in only 3 of 84 samples of different ethnicity. All the three reported subjects were of African American ethnicity, which is quite higher than our study group. Interestingly, they reported a novel SNP C1220A in 3 of 48 subjects with Asian ethnicity with 6% minor allele frequency [13]. These findings are contradictory to our results. On the basis of the genotypic analysis of our findings, we could not find any relationship between rs78202224 (C>A), rs139496713 (C>T) and rs45504694 (C>A) SNPs in HSF1 and HSF2 genes and male infertility. However, a association of rs78202224 of HSF1 was observed by Almotwaa et al. (2018) with breast cancer in Saudi females. On the other hand, Bridges et al. (2015) described 34 variants in the exonic sequence of human HSF1 gene and tried to analyse their biological consequences in human diseases [13,17]. Mou et al. (2013) identified three synonymous and five missense mutations of HSF2 gene in IA patients. Study demonstrated that the mutant genotype of HSF2 (R502H) suppressed the transcriptional regulatory function of the wild type allele through a dominant-negative effect and might be involved in human spermatogenesis failure. It suggested further implication of HSF2 as a potential therapeutic target [9]. These studies are in contrary to our observations.

Interestingly, both allelic and genotype association analysis revealed that the rs116868327 (G/A) variant in UBE2I gene is significantly associated with asthenozoospermia. Moreover, the genotype distribution between cases and controls also revealed that heterozygous condition at rs116868327 (G/A) locus is associated with an increased risk of male infertility [Odds ratio= 6.91, Confidence interval at 95%= 1.52-31.46, P value= 0.017]. Moreover, the allelic association analysis depicted that allelic distribution at rs116868327 locus in UBE2I gene differed significantly between cases and controls [Odds ratio= 6.75, Confidence interval at 95%= 1.50-30.49, P value= 0.018]. Similarly, some previous studies also depicted the role of UBE2I gene polymorphism in breast tumour progression. These studies concluded that women carrying the rare allele for rs7187167 in UBE2I gene showed an increased risk of grade 1 breast tumours [21,22]. Selection of variants for large-scale cohort analysis was a big challenge. While common variants may not be the risk factor for the disease and rare variants may not be present in the population at all. Accepting all the common variants and ignoring the rare variants may not be the way ahead. Therefore, we used a filtration method to find out the variants of interest. SNPs based on SIFT and Polyphen scores provided the work for future perspective.

## Conclusion:

Variation at rs116868327 locus in UBE2I gene increases the risk of asthenozoospermia. Allelic association analysis suggested that rare allele frequency was more in cases associated with the risk of asthenozoospermia in infertile males. However, none of the SNPs in HSF1 and HSF2 genes is linked with infertility in men in the study cohort. However, it is known earlier that HSF1, HSF2 and UBE2I genes are essential regulators for spermatogenesis. Thus, a representative analysis of variants/mutations of HSF1, HSF2 and UBE2I genes in multiple cohorts along with their functional assay will provide insights into the cause of male infertility.
